# Hepatoprotective effect of sitagliptin against methotrexate induced liver toxicity

**DOI:** 10.1371/journal.pone.0174295

**Published:** 2017-03-23

**Authors:** Hany M. Abo-Haded, Mohamed A. Elkablawy, Zeyad Al-johani, Osama Al-ahmadi, Dina S. El-Agamy

**Affiliations:** 1 Cardio-genetic team, College of Medicine, Taibah University, Almadinah Almonawarah, Saudi Arabia; 2 Department of pathology, College of Medicine, Taibah University, Almadinah Almonawarah, Saudi Arabia; Department of Pathology, Faculty of Medicine, Menoufia University, Menoufia, Egypt; 3 Department of Pharmacology and Toxicology, College of Pharmacy, Taibah University, Almadinah Almonawarah, Saudi Arabia; Department of Pharmacology and Toxicology, Faculty of Pharmacy, Mansoura University, Mansoura, Egypt; National Institutes of Health, UNITED STATES

## Abstract

Sitagliptin is selective dipeptidyl peptidase-4 inhibitor (DPP4-I), used clinically as a new oral anti-diabetic agent. This study explored the underlying mechanisms of the hepatoprotective role of sitagliptin pretreatment against methotrexate (MTX) induced hepatotoxicity in mice. Forty mice were divided into four groups (10 mice each); control, MTX, and two sitagliptin groups (pretreated with sitagliptin 10 and 20 mg/kg/day, respectively) for five consecutive days prior to MTX injection. Results showed that MTX induced marked hepatic injury in the form of cloudy swelling, hydropic degeneration, apoptosis and focal necrosis in all hepatic zones. Biochemical analysis revealed significant increase in the serum transaminases, alkaline phosphatase and lactate dehydrogenase in MTX group. Oxidative stress and depressed antioxidant system of the hepatic tissues were evident in MTX group. MTX down-regulated nuclear factor erythroid 2-related factor 2 (Nrf2) expression and reduced its binding capacity. Additionally, MTX increased the activation and the expression of nuclear factor kappa-B (NF-κB) and downstream inflammatory mediators. MTX induced the activation of inducible nitric oxide synthase (iNOS) and increased nitrite/nitrate level. Finally, hepatic cellular apoptosis was clearly obvious in MTX-intoxicated animals using TUNEL staining. Also, there was increase in the immunoexpression of pro-apoptotic protein Bax, increase in Bax and caspase-3 levels and decrease in the level of anti-apoptotic Bcl2 in liver. On the other hand, sitagliptin pretreatment significantly ameliorated all of the above mentioned biochemical, histopathological, immunohistochemical changes induced by MTX. These results provide new evidences that the hepatoprotective effect of sitagliptin is possibly mediated through modulation of Nrf2 and NF-κB signaling pathways with subsequent suppression of inflammatory and apoptotic processes.

## Introduction

Methotrexate (MTX) is a folic acid antagonist that has been widely used for the treatment of several malignancies, multiple sclerosis, dermatomyositis, sarcoidosis, psoriasis, rheumatoid arthritis and various inflammatory diseases [1,2]. Since the cytotoxic effect of MTX is not selective for the cancer cells, it can affect the normal tissues and so prolonged use of MTX has been associated with various organ toxicity. Clinically, hepatotoxicity remains one of the significant restrictions on its use in the doses desired. Previous reports have demonstrated the pivotal role of oxidative stress in MTX hepatotoxicity [3,4]. Excessive generation of reactive oxygen and nitrogen species (ROS/RNS), along with reduced antioxidant defense mechanism promote the development and progression of hepatotoxicity [5]. Recent studies have shown the possible role of inflammatory cytokines such as tumor necrosis factor-alpha (TNF-α) and inducible nitric oxide synthase (iNOS) in mediating MTX-induced hepatotoxicity [6]. Other studies have focused on the implication of nuclear factor kappa-B (NF-κB) pathway signaling in modulating MTX-induced hepatotoxicity [7].

Sitagliptin is a new oral glucose lowering agent that is currently used for the treatment of type II diabetes mellitus. It inhibits the dipeptidyl-peptidase IV (DPP-4) enzyme thereby prolonging the post-prandial activity of glucagon-like-peptide-1 (GLP-1) [8]. DPP-4 is widely distributed in all body organs and has pleiotropic biological activities. It is believed that DPP-4 is responsible for the modification of a number of regulatory factors including peptides or chemokines and affects the signaling functions. This suggests that DPP-4 is involved in determining immune responses and procession of inflammatory disorders as well [9]. Recently, the anti-inflammatory action of sitagliptin has been reported in different types of cardiovascular injury [10–12]. Notably, sitagliptin has shown hepatoprotective activity in experimentally induced steatohepatitis via modulation of lipid metabolism, oxidative stress and inflammatory mediators [13,14]. However, the exact mechanism of its hepatoprotective activity is complex and not completely clarified. To the best of our knowledge, the effect of sitagliptin against MTX-induced hepatotoxicity has not been tested. The present study tried to elucidate the possible hepatoprotective activity of sitagliptin against MTX-induced hepatotoxicity and its impact on different signaling pathways.

## Materials and methods

### Chemicals

Januvia tablets (highly water soluble sitagliptin phosphate monohydrate) and MTX ampoules (Hospira UK) were supplied by the Medical Center of Taibah University, Saudi Arabia. Sitagliptin tablet was dissolved in normal saline. Other chemicals were of highest grade and obtained from standard commercial supplies.

### Animals and experimental design

Forty male Swiss albino mice weighing 20 ± 2 g, were obtained from King Abdul-Aziz University, Jeddah, Saudi Arabia. Mice were housed in standard conditions of temperature, humidity and dark/light cycle. They were given standard laboratory rodent diet and tap water. All experimental procedures have been approved by “Research Ethics Committee of Taibah University, Saudi Arabia” which follows the Saudi National Regulation of the National Bioethics Committee, U.S. CFR and the Principles of Laboratory Animal Care.

Mice were randomly divided into four groups (10 mice per group) as follows:

Group 1: Control group where mice received normal saline until termination of the experiment.
Group 2: MTX group, was given a single injection of MTX (20 mg/kg, i.p.) on the sixth day.
Groups 3, 4: Sita+MTX groups, received sitagliptin at two different doses (10, 20 mg /kg) once daily for 5 consecutive days and single injection of MTX (20 mg/kg, i.p.) on the sixth day.

In a preliminary study, the effect of administration of sitagliptin (20 mg/kg) once daily for 5 consecutive days on the hepatic function and histopathology was estimated. This experimental group served as Sita group and it was compared to control mice.

On the seventh day, the animals were anesthetized by inhalation of diethyl ether and blood samples were collected from the retro-orbital plexus and allowed to clot. The serum samples were obtained and kept at -80°C until needed. Mice were then sacrificed by overdose of diethyl ether anesthesia with maximal effort done to minimize pain. Liver tissues were dissected and washed with ice-cold saline. Liver homogenates were obtained by homogenization in phosphate-buffered saline (PBS, pH 7.4) then centrifuged to get the supernatants which were kept at -80°C until analyzed. An extra sample of liver was excised and fixed in 10% neutral buffered formalin solution for histopathological and immunohistochemical analysis.

### Serum glucose level

It was estimated using glucose assay kit (Cell Biolabs, Inc., San Diego, CA, USA). The results were expressed as mg/dL.

### Serum indices of hepatotoxicity

Serum activities of alanine aminotransferase (ALT), aspartate aminotransferase (AST), alkaline phosphatase (ALP) and lactate dehydrogenase (LDH) were determined according to the protocol provided by colorimetric kits (Human, Wiesbaden, Germany) using spectrophotometer (UNICO Instruments C., Model 1200 USA).

### Liver histopathological examination

Paraffin blocks of livers were sectioned, stained with hematoxylin and eosin (H&E) and examined under a light microscope. The lesions were evaluated semi-quantitatively by ranking tissue lesion severity. Ranking from 0 to 3 depending on the degree and extent of the alteration as follows: (-) no pathological lesions, (+/-) very mild changes in < 5% of fields, (+) histopathology changes in < 20% of fields, (++) histopathology changes in 20 to 60% of fields, (+++) histopathology changes in >60% of fields. This ranking was used by Ragab et al. [15], to establish an overall assessment value of the histopathological lesion for studied animal tissues. Five high power fields were observed from each animal.

### Oxidative stress markers in liver tissue

Total antioxidant capacity (TAC), malondialdehyde (MDA), superoxide dismutase (SOD) and reduced glutathione (GSH) were measured in the liver homogenate using commercial kits (Bio-diagnostic, Giza, Egypt).

### Detection of liver NF-κB pathway by western blotting

Total 100 mg of liver tissue was homogenized in RIPA buffer (containing 1 μM phenylmethanesulfonyl fluoride) and then centrifuged (15000 × g, 5 min). Supernatants were collected and proteins were separated via an 8% SDS-PAGE (80 V, 30 min for concentrating gel; 120 V, 60 min for separating gel). Proteins were then transferred to a cellulose acetate membrane, blocked with 5% non-fat milk in buffer (20 mM Tris HCl, pH 7.4, 135 mM NaCl, 0.1% Tween) (2 h, at room temperature). Membranes were then incubated overnight (at 4°C) with the primary antibodies (NF-κB p65, p-p65, p-IKKα, p-IκBα; 1:200), followed by the secondary antibodies: anti-mouse (1:200) (1 h, at room temperature). Immunoreactive proteins were detected using DAB immuno-detection system. The size and intensity of the bands were analyzed. β-actin was used as an internal control.

### Liver immunohistochemical staining of NF-κB p65 and Bax

Mini tissue core (5 mm) was extracted from each paraffin liver block and used to construct a tissue miniarray (TmA) as previously described [16]. IHC staining of NF-κB p65 and Bax was carried out automatically using Ventana Bench Mark XT system (Ventana Medical Systems, Tucson, AZ) as reported before [17].

### Detection of apoptosis using TUNEL staining

TUNEL assay was performed on 4-μm slices of paraffin-embedded TmA liver tissue sections after xylene deparaffinization and hydration in graded ethanol according to the manufacturer's protocol. The stained apoptotic cells and bodies were identified using In Situ Cell Death detection kit (Roche Molecular Biochemicals, Switzerland). Apoptotic index (AI) data were presented as the average results of ten random fields examined from TmA slide of each group. The TUNEL-positive brown apoptotic hepatocytes nuclei and apoptotic bodies were counted and the data were expressed as the number of TUNEL-positive cells/high- power field (× 400) across 10 different fields for each TmA section.

### ELISA assay

TransAM Nrf2 ELISA kit was used to estimate active form of transcription factor nuclear factor erythroid 2-related factor 2 (Nrf2) in the hepatic nuclear extract following the manufacturer protocol (ActiveMotif, Carlsbad, CA, USA). Mouse Bax was determined using a kit obtained from MyBioSource Inc., San Diego, CA, USA. Nitrite/nitrate, tumor necrosis factor alpha (TNF-α), interleukin-1 beta (IL-1β), interleukin-6 (IL-6) and caspase-3 were measured using specific ELISA kits (R&D systems, Minneapolis, USA). Bcl-2 was determined using kit from Abbexa, Cambridge, UK.

### RT-PCR analysis

Gene expression analysis of Nrf2, iNOS, TNF-α, IL-1β, and IL-6 were determined in mice liver. Total RNA was isolated from mouse liver using RNeasy Mini kit (Qiagen, USA). The quality of the RNA were quantified spectrophotometrically using the ratio A260/A280 and it was used to select the pure RNA samples. RNA was reverse-transcribed to cDNA (Quantitect Reverse Transcription Kit, Qiagen, USA) and quantitative real-time PCR was done on thermocycler Rotor-Gene Q (Qiagen, Hilden, Germany) using SYBR Green (Qiagen, USA) and following the manufacturer protocol. The primers sequences used for the estimation of the selected gene were as follows: Nrf2 (Mouse) 5′-CCTCGCTGGAAAAAGAAGTG-3′ (sense); 5′-GGAGAGGATGCTGCTGAAAG-3′ (antisense), iNOS (Mouse) 5′-CGAAACGCTTCACTTCCAA-3′ (sense); 5′-TGAGCCTATATTGCTGTGGCT-3′ (antisense), TNF-α (Mouse) 5′-AAGCCTGTAGCCCACGTCGTA-3′ (sense); 5′-AGGTACAACCCATCGGCTGG-3′ (antisense), IL-1β (Mouse) 5′-TGGACGGACCCCAAAAGATG-3′ (sense); 5′-AGAAGGTGCTCATGTCCTCA-3′ (antisense), IL-6 (Mouse) 5′-ATGAAGTTCCTCTCTGCAAGAGACT-3′ (sense) 5′-CACTAGGTTTGCCGAGTAGATCTC-3′ (antisense), β-actin (Mouse) 5′- TCTACGAGGGCTATGCTCTCC-3' (sense); 5′-GGATGCCACAGGATTCCATAC-3′ (antisense).

The relative expression of different genes was determined by using the ΔΔCt method. Levels of Nrf2, iNOS, TNF-α, IL-1β, and IL-6 mRNA were normalized relative to β-actin (housekeeping gene) in the same sample. Rotor-Gene Q Software 2.1 (Qiagen) was used for data analysis.

### Statistical analysis

It was done using one way analysis of variance (ANOVA) followed by Tukey's Kramer Multiple Comparison Test. The values are means ± SEM for ten mice in each group. P value < 0.05 was considered as significant.

## Results

Data have shown no significant difference in the biochemical indices of hepatotoxicity or histopathological analysis of liver between animals of Sita group which received sitagliptin alone at (20 mg/kg) compared to that of the control group. That is why this group was further excluded to facilitate data comparison and interpretation (S1 File).

### Effects on serum glucose level and indices of hepatotoxicity

As shown in Table 1, serum glucose level was not significantly altered among different experimental groups. MTX treatment increased the serum activities of ALT, AST, ALP and LDH compared to the control group. Pretreatment with sitagliptin caused a significant decrease in liver transaminases, ALP and LDH levels compared to MTX group in a dose dependent manner (Table 1).

**Table 1:** Effects of Methotrexate (MTX) and Sitagliptin (Sita) on blood glucose level and biochemical parameters of liver function.

Groups	Glucose (mg/dL)	ALT (IU/L)	AST (IU/L)	ALP (IU/L)	LDH (IU/L)
Control	94.2 ± 5.4	32.6 ± 3.8	53.7 ± 5.0	28.1 ± 2.5	83.7 ± 6.9
MTX	109.1 ± 4.9	209.3 ± 22.1 ***	317.6 ± 30.1 ***	196.7 ± 16.8 ***	408.2 ± 25.6 ***
Sita (10 mg/kg) + MTX	103.3 ± 6.4	138.7 ± 5.4 ***^###^	208.5 ± 12.1 ***^###^	161.3 ± 5.2 ***^#^	342.5 ± 11.7 ***^#^
Sita (20 mg/kg) + MTX	96.1 ± 4.1	97.6 ± 4.9 **^###^	159.0 ± 14.4 **^###^	61.3 ± 3.8 ^###^	194.5 ± 9.2 ***^###^
Sita (20 mg/kg)	90.9 ± 6.8	36.5 ± 2.7	50.3 ± 3.1	25.1 ± 1.8	91.9 ± 6.1

n = 10.

**P < 0.01,

***P < 0.001 vs. the control.

^#^P < 0.05,

^###^P < 0.001 vs. the MTX group (ANOVA followed by Tukey-Kramer multiple comparison).

### Histopathological analysis of liver

**Fig 1:**
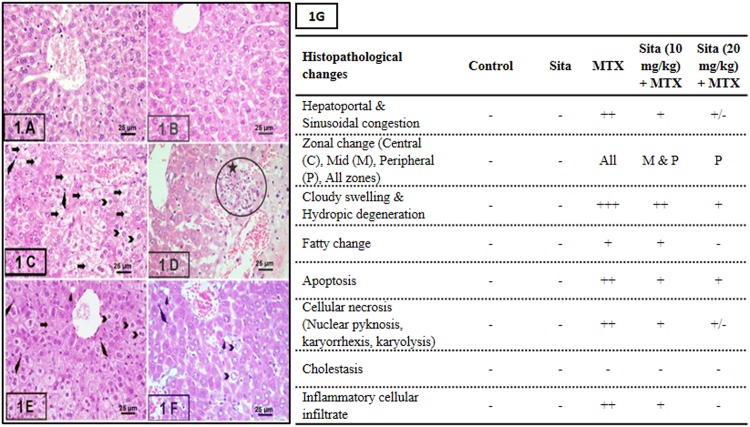
Sitagliptin (Sita) pretreatment ameliorates Methotrexate (MTX)-induced histopathological lesions in liver of mice (H&E stain, 400×). A. Control group showing normal liver histology, B. Sita group showing normal liver histology, C. MTX group showing cloudy swelling, hydropic degeneration (Chevron), fatty change (Arrow heads), shrinked apoptotic cells with pyknotic small nuclei and esinophic cytoplasm (wavy arrows) and focal necrosis (Arrows) in all hepatic zones. Many hepatocytes show signs of necrosis (Arrows) (pyknosis, kariorrhexis and karyolysis), D. Portal area of MTX group showed the periportal inflammatory cellular infiltrate (circle with star), E. Sita (10 mg/kg) + MTX group showing reduction in the histopathological lesions in the central hepatic lobular zone, F. Sita (20 mg/kg) + MTX group showing a significant reduction in the histopathological lesions in all hepatic lobular zones. G. Semi-quantitative scoring showing the hepatoprotective effects of Sita.

The histopathological changes and the semi-quantitative scoring are shown in Fig 1 Liver sections from control group and sita group exhibited normal liver architecture while mice of the MTX group showed a significant increase in the severity of necrosis and inflammation compared to the control group (Fig 1G). Histological examination showed the presence of cloudy swelling, hydropic degeneration, apoptosis and focal necrosis in all hepatic zones in MTX group. Pretreatment with sitagliptin resulted in a significant reduction in the MTX-induced pathological lesions in liver tissue with marked improvement in the fourth group.

### *Effects* *on hepatic oxidative stress markers*

Table 2 reflects the significant increase in oxidative stress in MTX group. MTX induced elevation in MDA content and depressed TAC, GSH and SOD in the hepatic tissue. Sitagliptin pretreatment alleviated markers of oxidative stress and improved the antioxidant capacity of the liver.

**Table 2:** Effects of Methotrexate (MTX) and Sitagliptin (Sita) on oxidative stress markers in the liver.

Groups	Parameters			
	TAC (mmol/g tissue)	MDA (nmol/g tissue)	SOD (Units/g tissue)	GSH (μmol/g tissue)
**Control**	0.8 ± 0.04	25.2 ± 1.7	16.3 ± 1.4	10.8 ± 1.2
**MTX**	0.4 ± 0.03 ***	49.1 ± 3.9 ***	6.0 ± 0.1 ***	5.3 ± 0.1 ***
**Sita (10 mg/kg) + MTX**	0.5 ± 0.03 ***	46.1 ± 3.9 ***	7.3 ± 1.1 ***	7.7 ± 0.2 *
**Sita (20 mg/kg) + MTX**	0.6 ± 0.03 *^###^	33.3 ± 2.8 ^##^	12.7 ± 0.9 ^###^	9.5 ± 0.8 ^##^

n = 10.

* P<0.05,

*** P<0.001 vs. the control.

^##^P<0.01,

^###^ P<0.001 vs. the MTX group (one-way ANOVA followed by Tukey-Kramer multiple comparison)

### Effects on Nrf2 expression

**Fig 2:**
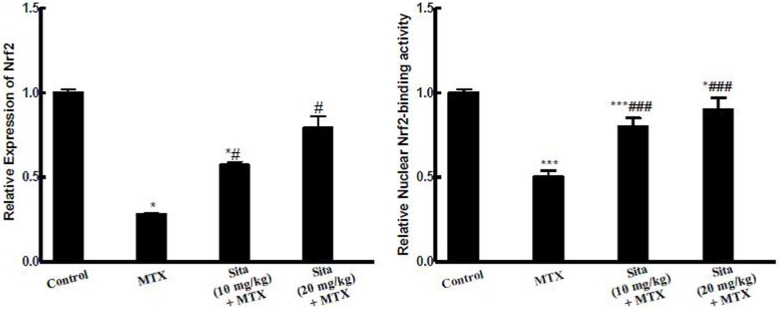
Sitagliptin (Sita) pretreatment improves Methotrexate (MTX)-induced reduction in Nrf2 expression and binding activity in the hepatic tissue of mice. n = 10. *P < 0.05, ***P < 0.001 vs. the control. ^###^P < 0.001 vs. the MTX group (ANOVA followed by Tukey-Kramer multiple comparison).

MTX injection down-regulated Nrf2 expression and reduced Nrf2 binding activity in the liver of MTX-intoxicated mice. This depression of Nrf2 activity may be correlated to the depression of the anti-oxidant status of the liver. Sitagliptin pretreatment up-regulated Nrf2 mRNA and restored Nrf2 binding activity compared to MTX group (Fig 2).

### Effects on NF-κB pathway

**Fig 3:**
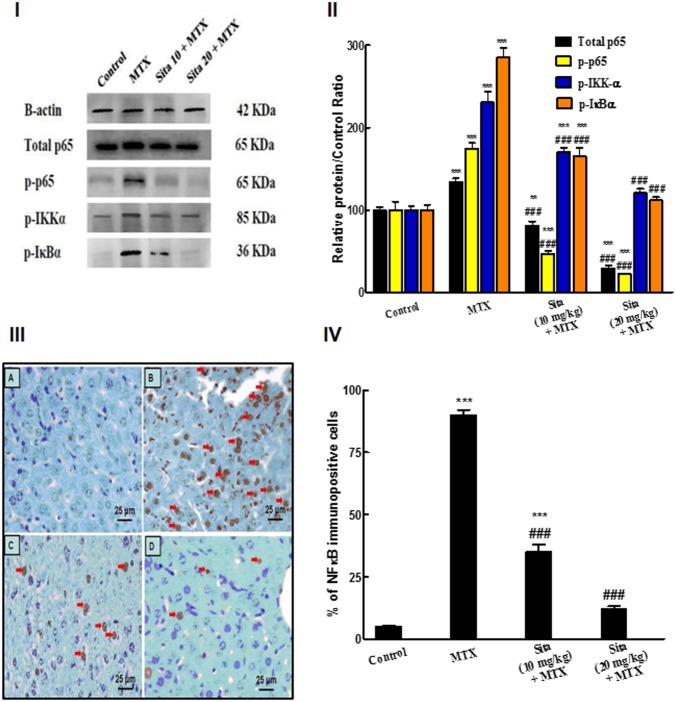
Sitagliptin (Sita) pretreatment suppresses Methotrexate (MTX)-induced activation of nuclear factor kappa-B (NF-κB) pathway in the hepatic tissue of mice. **I.** Western Blot for NF-κB p65, p-p65, p-IKKα, p-IκBα. **II.** Quantification of the protein intensities of relative protein level of NF-κB p65, p-p65, p-IKKα, p-IκBα. β-actin served as internal control. **III.** Immunohistochemical staining of nuclear NF-κB p65 (400×): A. Control group with focal light brown immunostaining, B. MTX group, showing intense brown immunostaining of NF-κB (Red arrows) (C, D) Sitagliptin + MTX groups, showing a marked decrease in the expression of NF-κB. **IV.** Semiquantitative analysis of NF-κB immunohistochemical staining results in liver tissues of different groups, expressed as % of NF-κB immunopositive cells across 10 different fields for each TmA section. n = 10. **P < 0.01, ***P < 0.001 vs. the control. ^###^P < 0.001 vs. the MTX group (ANOVA followed by Tukey-Kramer multiple comparison).

Western blot results indicated that hepatic tissue of MTX treated mice group exhibited activation of NF-κB pathway. MTX significantly increased total p65 and induced a significant enhancement in the phosphorylation of p65 compared to the control group. However, sitagliptin pretreatment significantly ameliorated total p65 as well as MTX-induced phosphorylation of p65 in the liver tissue compared to MTX group. For further confirmation, the phosphorylation status of IκBα was estimated. MTX induced a significant increase in p-IκBα compared to control group while sitagliptin pretreated groups showed significant reduction in the phosphorylation of IκBα with respect to MTX group. Additionally, IKKα phosphorylation was elevated in MTX group compared to control group. However, sitagliptin pretreatment significantly reduced the p-IKKα at both doses compared to MTX group (Fig 3I and 3II). These results were further validated by IHC analysis of hepatic tissue. The control group showed minimal brown nuclear immunostaining for NF-κB p65 (Fig 3III). MTX injection induced a highly significant increase in nuclear NF-κB p65 level in liver cells compared to control (Fig 3IV). Pretreatment of MTX intoxicated animals with sitagliptin at both lower and higher doses markedly decreased the NF-κB p65 expression (Fig 3III and 3IV).

### Effects on inflammatory cytokines

**Fig 4:**
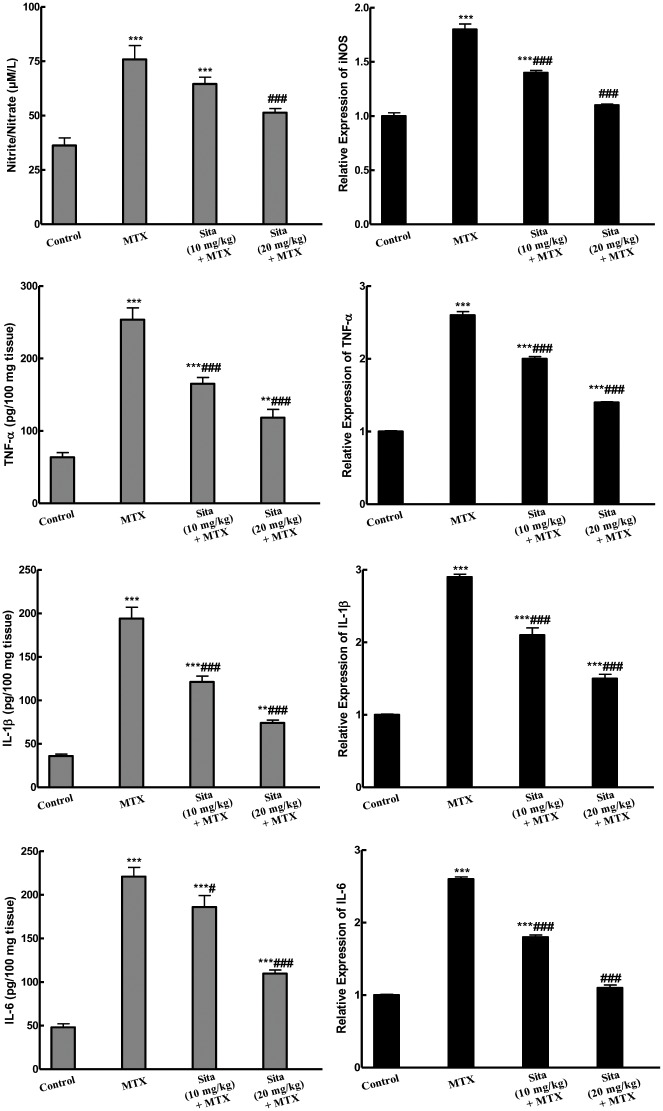
Sitagliptin (Sita) pretreatment alleviates Methotrexate (MTX)-induced increase in the levels and the expression of different inflammatory cytokines in the hepatic tissues of mice. n = 10. **P < 0.01, ***P < 0.001 vs. the control. ^#^P < 0.05, ^###^P < 0.001 vs. the MTX group (ANOVA followed by Tukey-Kramer multiple comparison).

As presented in Fig 4, MTX injection caused an increase in the expression of iNOS as well as the level and the expression of different inflammatory cytokines (nitrite/nitrate, TNF-α, IL-1β, IL-6). Sitagliptin pretreatment succeeded to counteract this increase and restored their normal levels.

### Effects on apoptosis

**Fig 5:**
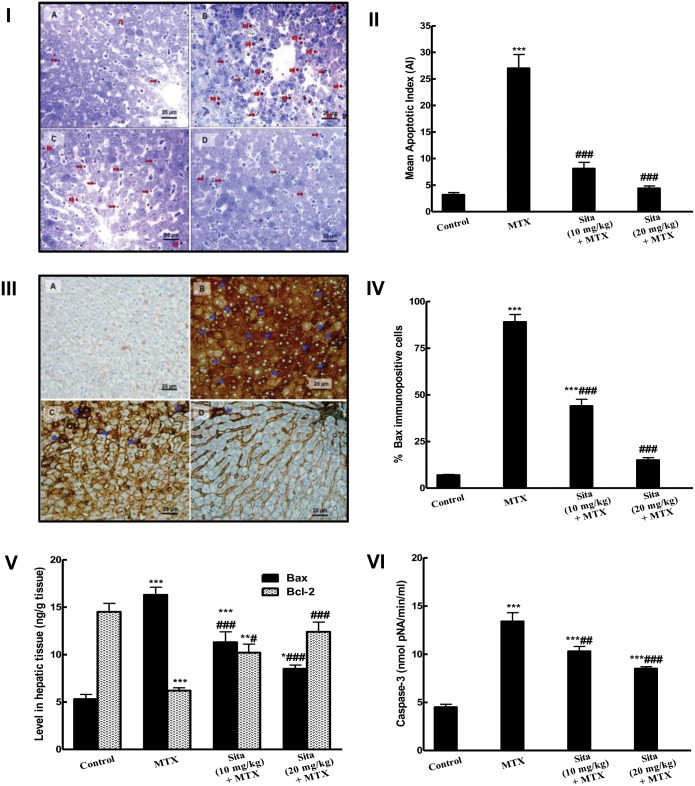
Sitagliptin (Sita) pretreatment counteracts apoptosis and improves apoptotic markers in the hepatic tissues of Methotrexate (MTX)-treated mice. I. TUNEL-positive apoptotic liver cells nuclei (Thick red arrow) and bodies (Thin red arrow) detected in the liver sections were: (A) Occasionally seen in Control mice group; (B) Frequently observed in mice group intoxicated with MTX; (C&D) Clearly decreased in sita pretreated groups with more marked reduction in the number of apoptotic cell and bodies seen in group pretreated with sita 20 mg/kg. II. Effect on mean apoptotic index (the number of TUNEL-positive cells/high- power field (× 400) across 10 different fields for each TmA section). III. The expression of pro-apoptotic marker Bax by immunohistochemical staining (400×): A) Control group with focal light brown cytoplasmic immunostaining in less than 1% of cells; B) MTX group, showing intense brown cytoplasmic immunostaining of Bax (Blue arrows) in more than 95% of cells (C, D) Sita + MTX groups, showing a marked decrease in the cytoplasmic expression of Bax in a dose dependent manner of sita. IV. Semiquantitative analysis of Bax immunohistochemical staining results in liver tissues of different groups, expressed as % of immunopositive cells in TmA sections of all animals of each group, 10 different fields/section. V. Levels of Bax and Bcl2 in hepatic tissue. VI. Caspase activity in hepatic tissue. n = 10. *P < 0.05, **P < 0.01, ***P < 0.001 vs. the control. ^#^P < 0.05, ^##^P < 0.01, ^###^P < 0.001 vs. the MTX group (ANOVA followed by Tukey-Kramer multiple comparison).

TUNEL assay provided an evidence of apoptosis in liver tissues. TUNEL-positive cells and apoptotic bodies appeared occasionally in the liver sections of control mice but they were frequently observed in MTX group (Fig 5I). TUNEL-positive apoptotic cells and bodies were reduced by sitagliptin administration at both lower and higher doses (Fig 5I). A highly significant difference was detected regarding AI between the MTX and the control groups. The AI in the MTX intoxicated animals pretreated with sitagliptin groups were significantly reduced compared to MTX group (Fig 5II). As shown in Fig 5III and 5IV, the immuno-expression and the level of the pro-apoptotic marker Bax were markedly increased in the hepatic tissue upon MTX administration compared to the control group. However, sitagliptin pretreatment significantly decreased Bax immuno-expression compared to the MTX group. This was further confirmed by the biochemical estimation of Bax level in hepatic tissues (Fig 5V). Additionally, the level of the anti-apoptotic marker Bcl2 was significantly decreased in the hepatic tissue of MTX intoxicated animals. This decrease was almost normalized in sitagliptin pretreated mice (Fig 5V). Caspase-3 activity was markedly increased in MTX treated animals while animal pretreated with sitagliptin showed significant lower level of caspase-3 compared to MTX group (Fig 5VI).

## Discussion

High doses of MTX, as used for acute leukemia or severe psoriasis have been associated with organ toxicity including acute hepatotoxicity, progressive hepatic fibrosis and cirrhosis [3,18]. Hereby, we tried to prove the potent hepatoprotective activity of sitagliptin against MTX-induced hepatic damage and we demonstrated that such protective effects are mediated through up-regulation of Nrf2 contaminant with down-regulation of NF-*κ*B with subsequent inhibition of inflammatory and apoptotic pathways.

Results of the present study showed that mice treated with MTX showed marked liver injury as indicated by significant increase in liver transaminases, ALP and LDH. These cytosolic enzymes are the best indicator of liver necrosis. Increase in their activities in the serum indicates a leakage in cell membrane, which in turn, is associated with hepatocyte death [6,7]. The biochemical changes were supported by histopathological examination results which revealed marked hepatic injury in MTX group. These biochemical and histopathological changes were significantly attenuated by sitagliptin pretreatment suggesting that sitagliptin could effectively counteract MTX -induced liver cell injury.

Previous reports have demonstrated the role of ROS/RNS in the pathogenesis of MTX-induced hepatotoxicity. These highly reactive species react with biological macromolecules producing lipid peroxides, inactivating proteins and mutating DNA. Results of the present study showed that MTX significantly altered the oxidant/antioxidant balance. MTX increased MDA level accompanied with depressed TAC, decreased GSH content and SOD activity. These results are in accordance with previous reports [3,5,19] which supports the hypothesis that oxidative cellular damage with profound lipid peroxidation are hallmarks of MTX toxicity [4,20]. The oxidative damage induced by MTX in liver tissue was prevented by sitagliptin pretreatment as it provided anti-oxidant effects not only on the non-enzymatic defense system (GSH), but also on the enzymatic one such as SOD. Similar antioxidant effects of sitagliptin were recently reported in many inflammatory conditions including myocardial injury [12,21], liver steatohepatitis [13,14], type 2 diabetes [22] and renal ischemia/reperfusion injury [23].

Nrf2 is a transcriptional activator that can serve as a sensor for oxidative stress. Under normal conditions, Nrf2 is retained in the cytoplasm with Kelch-like ECH associated protein (Keap1). In state of oxidative stress, Nrf2 signaling pathway is activated through its release from its cytoplasmic Keap1 and nuclear translocation with subsequent high Nrf2 activity. However, under extreme conditions of oxidative stress, Nrf2 remains within the cytoplasm [24]. Nrf2 has an important role in the regulation of defensive genes activation and induction of antioxidant enzymes as SOD, catalase (CAT) and glutathione peroxidase (GPx) leading to suppression of injury evoked by ROS and protection of cells against oxidative stress injurious effects [7,25]. Our results indicated the MTX induced a decrease in mRNA of Nrf2 and Nrf2 binding capacity. This can partially explain the depression of the anti-oxidant status of liver in MTX treated mice. On the other hand, sitagliptin pretreatment increased the expression of Nrf2 and increased its binding activity. This finding is in harmony with the recent study of Choi et al. [26] which reported the ability of gemigliptin, another DDP4 inhibitor, to exert protective vascular effects via enhancement of Nrf2 activity. The plausible mechanism for sitagliptin on Nrf2 might explain sitagliptin ability to increase SOD activity leading to enhancement of the antioxidant defense status of the liver and hence amelioration of oxidative stress. However, this point remains to be further explored.

NF-κB is a nuclear transcription factor that plays a pivotal role in the pathophysiology of MTX-induced hepatotoxicity. This factor is present in the cytoplasm in inactive state as it complexes with its inhibitor the IκBs (α or β). As a result of oxidative stress, IKKα or IKKβ phosphorylates IκBs leading to release of NF-κB and its translocation into the nucleus. Subsequently, NF-κB binds to DNA and up-regulates the transcription of many inflammatory genes like cytokine, chemokine and receptors of advanced glycation end products [27]. The present study revealed the marked activation of NF-κB pathway (NF-κB, p-IKKα and p-IκBα) upon MTX administration which is in accordance with the previous reports [7,28]. Sitagliptin pretreatment showed marked inhibitory effect on NF-κB activation pathway. These results are in line with previous reports which showed the ability of sitagliptin to counteract NF-κB activation [12,29]. Taken together, these results clearly suggests for the first time that sitagliptin hepatoprotective role may be closely linked to its ability to modulate NF-κB and Nrf2 cross-talk.

For better understanding of the molecular mechanism of the anti-inflammatory activity of sitagliptin, it was essential to evaluate its effects on the mRNA expression of inflammatory genes in MTX intoxicated animals. Previously, it was reported NF-κB activation leads to induction of cytotoxic cytokines which aggravate liver damage [30]. TNF-α is a proinflammatory cytokine playing a central role in mediating the inflammatory response. It induces other cytokines release as well as production of nitric oxide which enlarge oxidative damage [31]. Results have shown that expression and the release of TNF-α, IL-1β and IL-6 were increased in the hepatic tissue of MTX group while sitagliptin+MTX groups showed significant down-regulation of these elevated inflammatory markers. These results are in line with the previous ones which reported ability of sitagliptin to counteract LPS-induced release of different cytokines [29]. Similarly, the expression of iNOS and the level of nitrite/nitrate were increased in the hepatic tissue of MTX-intoxicated animals. The crucial role of NO in pathogenesis of the toxic effects of MTX is well documented in previous investigations. High levels of NO can react with superoxide anion to produce the potent and versatile oxidant peroxynitrite and induce the activation of NF-κB [28]. Sitagliptin pretreatment down-regulated iNOS expression and inhibited nitric oxide release. That inhibitory effect of sitagliptin against nitric oxide production is in agreement with previous studies [12].

Finally, apoptotic cell death was detected using of TUNEL assay which is a common standard method used to detect apoptotic cells. MTX-treated animals exhibited high percentage of TUNEL positive cells while sitagliptin pretreated groups showed marked protection against apoptotic cell death. Previous investigations have focused on the pivotal role of Bcl-2 family which includes pro-apoptotic protein (as Bax) and anti-apoptotic proteins (as Bcl-2 and Bcl-xL) as the major regulators of apoptosis. During MTX hepatotoxicity, increase of oxidative stress induces Bax translocation to the outer mitochondrial membrane resulting in increase in mitochondrial permeability and cytochrome c release into the cytosol which activate downstream effector caspases [7]. The results of the present study indicate that MTX induced obvious hepatocellular apoptosis as there was increase in Bax level and expression as well as caspase-3 activity in the hepatic tissue upon MTX administration with contaminant decrease in Bcl-2. These changes were significantly ameliorated by sitagliptin pretreatment. These results are in accordance with previous investigations that reported the ability of sitagliptin to suppress apoptosis through modulation of apoptotic and anti-apoptotic proteins [12,21,32]. Enhancement of antioxidant status of the liver through Nrf2 activation, inhibition of NF-κB with subsequent inhibition of inflammatory cytokines and reduction of apoptosis through control of apoptotic and anti-apoptotic proteins promote hepatocyte survival against MTX toxicity.

## Conclusions

**Fig 6:**
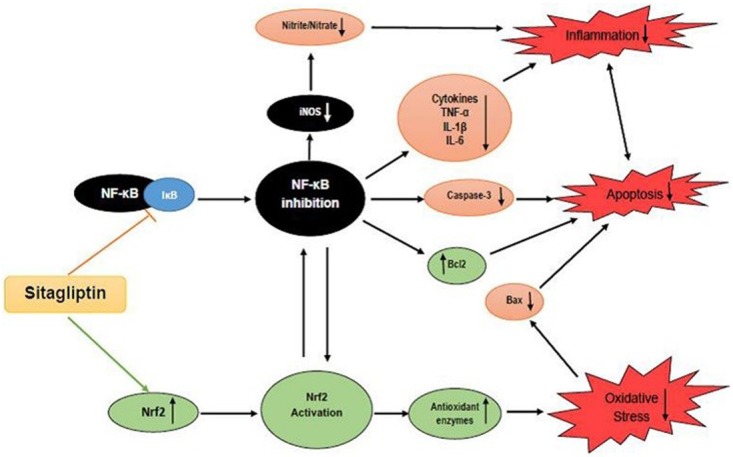
Schematic diagram summarizing the possible mechanisms for the hepatoprotective effects of Sitagliptin (Sita) against Methotrexate (MTX)-induced hepatotoxicity.

In light of all the previous findings, these data suggest that administration of sitagliptin may confer protection against MTX-induced hepatotoxicity which could possibly be ascribed, in part, to modulation of Nrf2 and NF-κB signaling pathways resulting in regulation of the oxidant/anti-oxidant balance, potent anti-inflammatory and anti-apoptotic activity (Fig 6). As sitagliptin has no serious side effects reported on its chronic use [33], it may be superior to other hypoglycemic agents in case of patients receiving MTX. Further investigations are needed to explore the other molecular pathways involved in sitagliptin hepatoprotective effect and to confirm that effect clinically.

## Supporting information

S1 File Effects of Sitagliptin (Sita) pretreatment on biochemical, histopathological, molecular indices of hepatotoxicity. (XLSX)
